# Reporting errors in infectious disease outbreaks, with an application to Pandemic Influenza A/H1N1

**DOI:** 10.1186/1742-5573-7-12

**Published:** 2010-12-15

**Authors:** Laura F White, Marcello Pagano

**Affiliations:** 1 Department of Biostatistics, Boston University School of Public Health, 801 Massachusetts Ave, 3rd Floor, Boston MA 02118 USA; 2 Harvard School of Public Health, 655 Huntington Ave, Boston MA 02115 USA

## Abstract

**Background:**

Effectively responding to infectious disease outbreaks requires a well-informed response. Quantitative methods for analyzing outbreak data and estimating key parameters to characterize the spread of the outbreak, including the reproductive number and the serial interval, often assume that the data collected is complete. In reality reporting delays, undetected cases or lack of sensitive and specific tests to diagnose disease lead to reporting errors in the case counts. Here we provide insight on the impact that such reporting errors might have on the estimation of these key parameters.

**Results:**

We show that when the proportion of cases reported is changing through the study period, the estimates of key epidemiological parameters are biased. Using data from the Influenza A/H1N1 outbreak in La Gloria, Mexico, we provide estimates of these parameters, accounting for possible reporting errors, and show that they can be biased by as much as 33%, if reporting issues are not accounted for.

**Conclusions:**

Failure to account for missing data can lead to misleading and inaccurate estimates of epidemic parameters.

## Background

The recent outbreak of pandemic strain Influenza A H1N1 (pH1N1), as well as other infectious disease outbreaks that have taken place recently illustrate the need for a rapid public health response and the ability to collect and analyze data efficiently. Unnecessary panic and disruption to society is more likely to be avoided and appropriately measured public health responses are more likely to take place when we have accurate information on the virulence and pathogenicity of an emerging disease. For these reasons, quantitative methods have been developed and continue to be developed to facilitate the assimilation of emerging data. Important quantities to estimate include the basic reproductive number, R_0_, defined as the average number of secondary infections created by a single infected individual in an entirely susceptible population. Numerous methods have been proposed for the estimation of this quantity, including deterministic and stochastic compartmental models [1], branching processes [2], networks [3,4], and, more recently, a likelihood based method [5,6].

Another parameter of interest is the serial interval, or the distribution of the interval in time between an infector and infectee presenting with symptoms [7,8]. It has been recently shown that this quantity may be time dependent; for example it can contract during the course of an epidemic as prevalence of the disease increases [9]. Other quantities of interest include the case fatality rate [10] and the attack rate in subpopulations, such as age groups.

Among methods that can be implemented with relatively straightforward data, we typically assume complete observations. Clearly this assumption is more often than not violated in practice, especially when dealing with national or even regional data. For instance the scare surrounding the anthrax attacks in the fall of 2001 in the United States led to a large number of individuals reporting to medical care facilities with suspected anthrax. Should analysis have focused on suspected cases in that situation, the magnitude and threat of the event would have been greatly exaggerated. During the recent H1N1 outbreak, the number of suspected cases of disease likely is composed of several cases that will not be confirmed, however there are undoubtedly an even larger number of undetected cases, at least in the initial stages of the epidemic before the large public health response was launched and later on as the growth of the epidemic in many locations rendered it impossible to continue to track a large portion of the cases. Further, as the pandemic progressed sick individuals were cautioned to stay at home, rather than seek medical attention unless they were acutely ill [11], driving up the number of unreported cases. Several of the unreported initial cases could arise from individuals who are asymptomatic, but still carrying and transmitting the virus or from others whose illness was not sufficiently acute to warrant seeking medical attention. To our knowledge, the issue of the impact of this misspecification of the number of cases on the estimation of epidemic parameters has not been well-studied.

In what follows we use the likelihood based methodology described in [5] to broach the subject and investigate the impact of underreporting on estimates of both R_0 _and the serial interval. First we provide an overview of the methodology we employ and introduce notation to describe the occurrence of misspecification of cases. Second we provide some theoretical results describing the impact on the estimation of the reproductive number. We illustrate this through a simulation study. Finally we investigate the impact that various plausible underreporting schemes would have on estimates obtained from the recent H1N1 outbreak in La Gloria, Mexico and compare these to estimates of R_0 _obtained using the method proposed by Wallinga and Teunis (WT method) [3] and a simple exponential growth model [12].

## Methods

In what follows, we assume that the outbreak is in its initial phase and that there is an unlimited supply of susceptible individuals. This implies that all contacts that an infected individual has are with susceptible individuals. Additionally, we follow standard methods and assume homogenous mixing among individuals in the system being studied. Following [5], we assume that **N***_t _*= {*N*_1_, ..., *N_T_*} are the number of new cases each day of the epidemic, with T being the total number of days of data analyzed, and that the serial interval is given by **p **= {*p*_1_, ..., *p_k_*} where k is the maximal length of the serial interval and *p_j _*is the probability of an infectee presenting with symptoms j days after the infector. In practice we can model the *p_j _*with a multinomial distribution or a truncated continuous distribution, such as the Gamma or Weibull, and estimate the parameters of that distribution so that the dimensionality of the estimation is independent of *k*. Then, we show in [5] that the likelihood of the case counts **N***_t _*= {*N*_1_, ..., *N_T_*} is given by a thinned Poisson:

(1) $$L(R_{0},p)=\prod_{t=1}^{T}\frac{e^{-\mu_{t}}\mu_{t}^{N_{t}}}{N_{t}!}.$$

where $\mu_{t}=R_{0}\sum_{j=1}^{\min(k,t)}N_{t-j}p_{j}$. Consider that on a given day *M_t _*= *q_t_N_t _*of the cases are observed, where *q_t _*≥ 0. Therefore little changes in (1) and it can be shown that the likelihood becomes

(2) $$L(R_{0},p,q)=\prod_{t=1}^{T}\frac{e^{-\mu_{t}}\mu_{t}^{\left(M_{t}/q_{t}\right)}}{(M_{t}/q_{t})!}.$$

where $\mu_{t}=R_{0}\sum_{j=1}^{min(k,t)}p_{j}M_{t-j}/q_{t-j}$. Given that *q_t _*is known, estimation proceeds as described in [5]. In reality we seldom known *q_t _*and our intent here is to quantify the effects of ignorance of *q_t _*on estimation of *R*_0 _and the serial interval.

### Estimation of *R*_0_

We first consider the case where the serial interval (the *p_j_*) is well known and specified, perhaps from contact tracing data or historical information. Then the MLE for *R*_0 _in the complete data case is given by

$${\hat{R}}_{0}=\frac{\sum_{t=1}^{T}N_{t}}{\sum_{t=1}^{T}\sum_{j=1}^{k}p_{j}N_{t-j}}$$

which is comparable to the branching process estimator described by [2], as illustrated in [5]. If *p_j _*is incorrectly specified we know that the estimates of *R*_0 _are impacted [5]. Our interest here is the study of the impact of missingness therefore we assume that *p_j _*is correctly specified so as to avoid confounding the effect of these two issues when estimating *R*_0_. In the case where data is incorrectly reported, the estimator for *R*_0 _obtained from [2] is

(3) $${\tilde{R}}_{0}=\frac{\sum_{t=1}^{T}M_{t}/q_{t}}{\sum_{t=1}^{T}\sum_{j=1}^{k}p_{j}M_{t-j}/q_{t-j}}.$$

We consider two simple missingness patterns in this scenario. First let the missingness be constant, i.e. *q_t _*= *q *for all t. Second let *q_t _*= *q*_1 _for *t *≤ *t_c _*and *q_t _*= *q*_2 _for *t *>*t_c_*, where *t_c _*might correspond to a public health announcement or certain number of cases occurring so as to raise alarm of an epidemic. In these cases it is likely that *q*_1 _<*q*_2_. This is the likely scenario initially in the current H1N1 outbreak, where cases were accumulating for some time before public announcements were made and increased surveillance was implemented. Numbers of confirmed cases available early on in the investigation likely underestimate the true number of cases dramatically. One can also imagine cases where *q*_1 _>*q*_2_. This might occur in instances of overreporting such as occur in times of panic, for instance following the scare after the anthrax attacks in 2001 in the US [13] when more than 20,000 individuals started antibiotics until it was determined that they did not have anthrax. Additionally if all suspected cases of H1N1 were considered early in the epidemic, this could possibly overestimate the true number of cases. Further, as time has progressed in the H1N1 pandemic, it has become virtually impossible to ascertain all cases. Therefore it is likely that reporting initially increased and then began to decrease again as case counts escalated.

We now provide results to illustrate the impact of these reporting schemes on the estimation of *R*_0_. In the following scenarios we use the branching process estimator to avoid the complication of the serial interval. In essence this implies that we assume that the serial interval is one day long in all scenarios or that the data is grouped into generations, rather than days or some other time unit. We now compare the two branching process estimators of the reproductive number, ${\hat{R}}_{0}$ and ${\tilde{R}}_{0}$ that make use of the observed numbers of cases denoted by **M***_t _*= {*M*_1_, ...., *M_T_*} where

$${\hat{R}}_{0}=\frac{\sum_{t=1}^{T}M_{t}}{\sum_{t=1}^{T}M_{t-1}},\;{\tilde{R}}_{0}=\frac{\sum_{t=1}^{T}M_{t}/q_{t}}{\sum_{t=1}^{T}M_{t-1}/q_{t-1}}.$$

In other words, ${\hat{R}}_{0}$ is the naïve estimator that does not account for reporting issues and ${\tilde{R}}_{0}$ is the true estimator. We prove the following two propositions in the Appendix.

**Proposition 1**. If *q_t _*= *q *for all values of t, then ${\hat{R}}_{0}$ and ${\tilde{R}}_{0}$ are equivalent.

**Proposition 2**. If *q_t _*= *q*_1_, *t *≤ *t_c _*and *q_t _*= *q*_2_, *t *>*t_c _*then

i. If *q*_1 _<*q*_2 _then ${\hat{R}}_{0}>{\tilde{R}}_{0}$, i.e. we overestimate *R*_0 _if naïve.

ii. If *q*_1 _>*q*_2 _then ${\hat{R}}_{0}<{\tilde{R}}_{0}$, i.e. we underestimate *R*_0 _if naïve.

In summary, if the reporting fraction does not change through time, then the estimate of R_0 _is unaffected. However if the reporting fraction increases (decreases) then we will overestimate (underestimate) R_0 _if we ignore misreporting (labeled naïve, above).

### Simulation study

In order to quantify the impact of missing data on the estimation of *R*_0 _and the serial interval, we consider a simulation study. We use multinomial and gamma distributed serial intervals. The multinomial represents a recent estimate for the current Influenza A/H1N1 outbreak in the USA and has a mean, *μ *of 2.21 days and variance, *σ*^2 ^of 0.89 with k = 4 [14]. The Gamma distributed serial interval represents a disease with a mean of 8 days and variance of 16 days with k set to 20. We consider three values for *R*_0_: 1.5, 2, and 2.5, making six simulation scenarios.

We then apply four missingness schemes to each dataset. The first two scenarios assume that the reporting fraction is constant through time. The second two schemes have the reporting fraction increase once 30 cases are accumulated. Following are the schemes that we consider:

1. *q_t _*= 0.1, for all t,

2. *q_t _*= 0.5, for all t,

3. *q*_1 _= 0.05, *q*_2 _= 0.5,

4. *q*_1 _= 0.4, *q*_2 _= 0.9.

Thus we have six sets of complete data and 24 sets of incomplete data, where each set of data has 10,000 simulated epidemics. We show results for data simulated with two initial cases (i.e. *N*_0 _= 2). Epidemics are simulated to stop when 500 cases are created or they die out. We only consider those that have at least ten cases for analysis, since fewer than ten cases would likely not be detected and considered an epidemic. Thus all simulated epidemics that die out before ten cases are accumulated, as well as those which have fewer than ten cases after the missingness pattern is applied, are not analyzed. Additionally those simulations with more than k zeroes in a row after the missingness operation is applied are not analyzed. This would be comparable to an undetectable epidemic since cases are so sparse in time that they are likely not connected to the same source.

**Figure 1:**
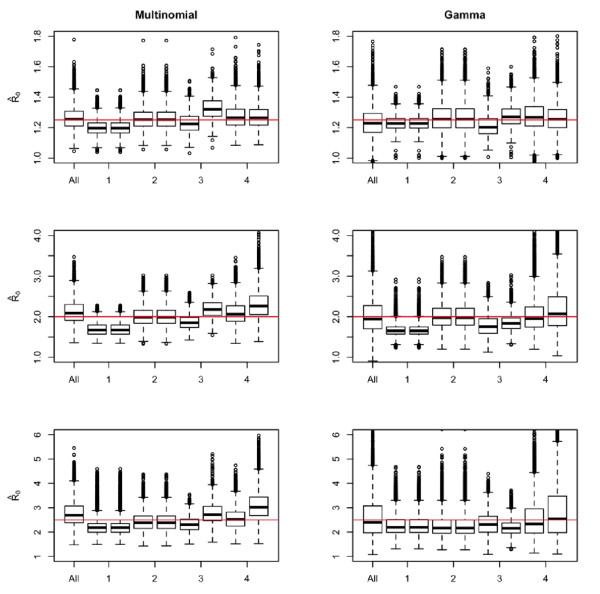
**Simulation results for the estimate of the reproductive number**. The first boxplot (All) in each frame shows results when all the data is used. Each subsequent couplet of boxplots first shows the estimates when missingness is correctly accounted for and second, when missingness is ignored when estimating. The numbers on the × axis denote the missingness scheme applied to the data (see text of manuscript for a detailed description). For example the first couplet corresponds to constant missingness of 0.05 with the left boxplot giving the results when missingness is accounted for and the right one for estimates when missingness is ignored. The horizontal line indicates the true value of the parameter.

**Figure 2:**
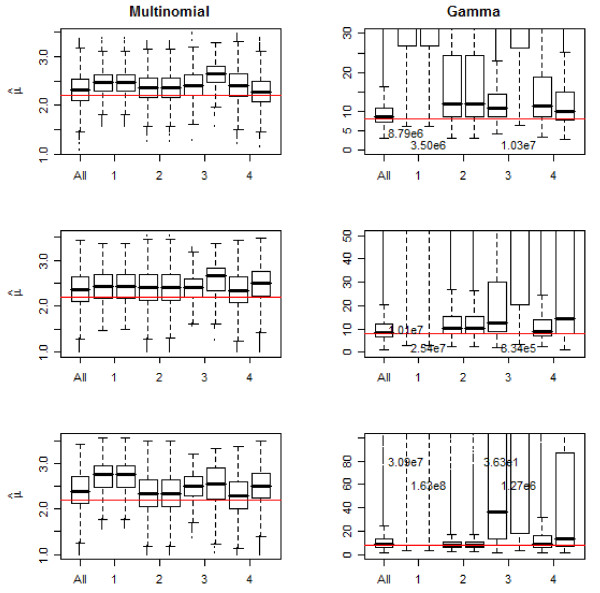
**Simulation results for the estimate of the mean of the serial interval**. The first boxplot in each frame shows results when all the data is used. Each couplet thereafter shows estimates when missingness is correctly accounted for on the left and on the right, when missingness is ignored in estimation. The numbers on the × axis denote the missingness scheme applied to the data(see text of manuscript for a detailed description). The horizontal line indicates the true value of the parameter. For several of the gamma distribution scenarios the actual estimates are extremely large and thus the value of the median is included on the plot where necessary.

The results of the simulations are given in Figure 1 and 2. Consistent with our theoretical results we observe that when the reporting fraction is constant, the estimates of R0 are unaffected by a failure to control for missingness. However if the reporting fraction increases, then the estimates are smaller when we adjust for the missingness. We also note that [15] has recently described a tendency of this method to overestimate the mean of the serial interval when the serial interval is short, such as in cases of influenza. Thus part of the effect seen could be attributed to this phenomena, but likely will be uniformly so.

### Influenza A/H1N1 in La Gloria, Mexico

**Figure 3:**
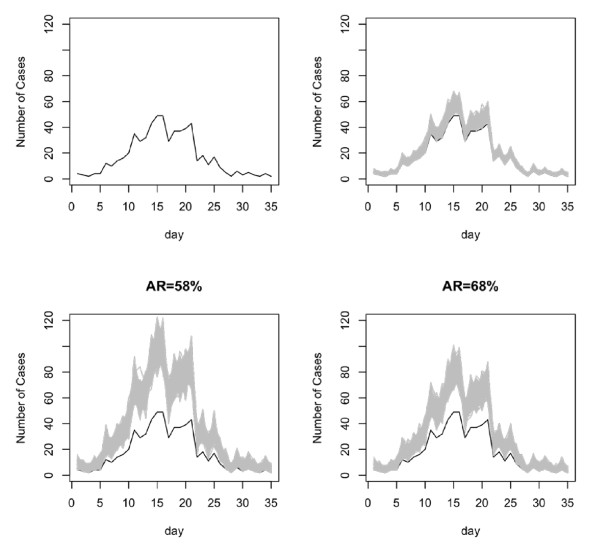
**Data simulated assuming constant reporting fraction**. The black curve shows the original data. The gray curves are the simulated datasets.

In [16] the authors report initial findings on the current H1N1 Influenza pandemic, including results from data collected on a localized outbreak in La Gloria, Mexico. Data for this analysis was obtained by surveying 1575 villagers out of 2243 villagers recorded in 2005 [17]. Of those surveyed, 615 cases were reported. It was later discovered that some of these reported cases were from seasonal flu. Figure 3 shows the observed data.

In addition to employing our likelihood based method (hereafter MLE method) to obtain estimates for the reproductive number and serial interval from the observed data, we also consider various schemes of underreporting among both those surveyed (due to asymptomatic cases or misclassification) and those not surveyed. Additionally we consider the possibility of overreporting, given that it was later noted that some of the cases reported were actually seasonal flu strains [18]. These reporting patterns are informed using the following pieces of information.

#### Attack Rate

Estimates of the attack rate for Influenza vary greatly. In [19] the authors report an attack rate of 68% among servicemen during an H1N1 outbreak in Finland during the winter of 1977-78. Among the 1575 surveyed in La Gloria an attack rate of 39% (615/1575) was observed. Finally, a recent report in Peru [20] indicates that 33% of cases were asymptomatic, meaning that the attack rate in La Gloria could actually be as high as 58% if we can extrapolate from Peru. The number of missing cases was calculated as a Poisson random variable with mean given by AR*(2243*AR-615). We generate 1000 epidemic sizes for each attack rate.

#### Constant reporting fraction

We first assume that there is a constant reporting fraction. In other words we attempt to study the impact of missing information on the individuals that were not surveyed, assuming that they would have followed the same trends as those who were surveyed. Using the simulated epidemic sizes, we superimpose the missed cases on the observed cases using a multinomial distribution with $q_{t}=N_{t}/\sum_{i=1}^{T}N_{i}$. The reporting fractions for attack rates of 39%, 58%, and 68% are 0.70, 0.53, and 0.40, respectively.

The data simulated from these assumptions are shown in Figure 3.

#### Varying reporting fraction

**Figure 4:**
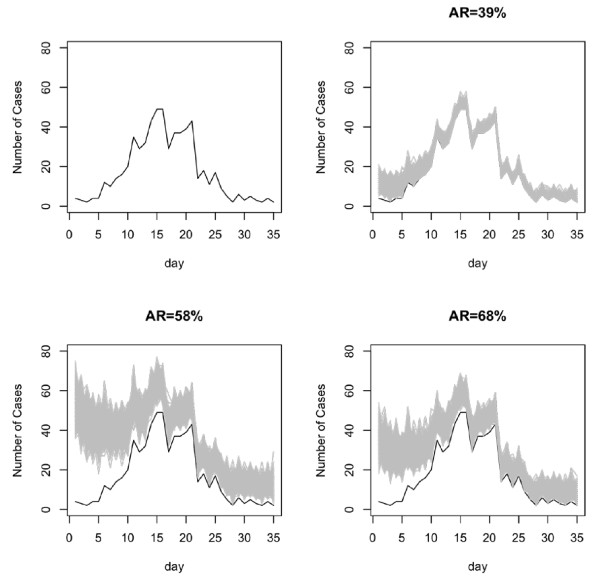
**Simulated data with logistic missingness where the reporting fraction increases with time**. The black curve shows the original data. The1 gray curves are the simulated datasets.

We consider three additional scenarios where we allow the reporting fraction to vary through time. In this case we let the reporting fraction follow a logistic function given by:

$$q_{t}=q_{2}q_{1}exp\left(\frac{rt}{q_{2}+q_{1}(e^{rt}-1)}\right)$$

where r describes the growth rate of the epidemic and is calculated at 0.19 in this scenario and q_1 _and q_2 _are set to 0.4 and 0.9, respectively and represent the reporting fractions at the start and end of the epidemic. Again the multinomial distribution with q_t_, as calculated from the logistic function, is used to assign the missing cases to days of the epidemic. Figure 4 illustrates the simulated outbreaks.

#### Overreporting

Finally we consider the possibility that a significant fraction of the cases were not actually pandemic influenza strain and, in fact, cases were overreported. We still assume that there was an overall underreporting of influenza like illness (ILI) according to the schemes described above. However, we additionally assume that only a fraction of those cases are indeed of the pandemic strain. This is done by randomly sampling from all of the ILI cases using a binomial distribution with probability 0.25, 0.50 or 0.75, indicating that 25%, 50% or 75% of cases, respectively, are pH1N1.

For the MLE analyses, we calculate the estimates of R_0 _and the serial interval, assuming the serial interval follows a multinomial with a maximal length of four days [14]. We estimate the parameters under two scenarios. First we consider the first 16 days when the epidemic curve is in exponential growth and 326 cases had been observed. We perform estimation assuming an infinite number of susceptible individuals. Second, we consider the entire epidemic curve and estimate the effective reproductive number R_t_, by allowing R_t _to following a four parameter logistic distribution, as shown in [6]. We report the value for R_0 _from this analysis. The median of the estimates over the 1000 simulated datasets is shown for each scenario along with the interquartile range of estimates obtained.

Additionally we provide estimates obtained on the same data where all cases are assumed to be pH1N1 using the method described by Wallinga and Teunis [3] for the entire dataset with either the serial interval estimate obtained from the MLE based method or that obtained by [16]. R_0 _is reported as the average R_t _over the first 16 days of the epidemic. We further use a simple exponential growth model to estimate the exponential growth parameter and R_0_, using both serial interval estimates [12].

## Results

Table 1 shows the results from the analysis for the MLE based method assuming that all the cases are pH1N1. The results that assume overreporting of pH1N1 are given in the appendix (Tables 2 and 3). We first consider the results obtained by considering the exponential growth phase of the epidemic (the first 16 days). Without any adjustments for underreporting, we estimate the basic reproductive number to be 1.41 and the mean of the serial interval to be 2.09 days with a variance of 1.15 days. When we assume constant missingness, the estimates for the reproductive number and the mean of the serial interval vary slightly from these estimates with their IQR containing the original values. Allowing the reporting fraction to vary according to a logistic function yields estimates of the reproductive number and the serial interval that are consistently less than the estimates from the original data.

**Table 1:** Results for the analysis of the La Gloria data.

		R_0 _(IQR)	$\hat{\mu }$**(IQR)**	${\hat{\sigma }}^{2}$**(IQR)**
All Data	Original	1.42	1.96	1.14
	
	39%	1.49 (1.44, 2.00)	2.15 (2.04, 2.47)	1.43 (1.27, 1.67)
	
	logistic	1.35 (1.32, 1.41)	2.11 (2.00, 2.22)	1.55 (1.35, 1.75)
	
	58%	1.55 (1.46, 2.03)	2.25 (2.08, 2.49)	1.45 (1.23, 1.67)
	
	logistic	1.13 (1.12, 1.15)	1.80 (1.70, 1.90)	1.32 (1.09, 1.53)
	
	68%	1.54 (1.46, 2.02)	2.24 (2.07, 2.48)	1.44 (1.23, 1.66)
	
	logistic	1.08 (1.07, 1.09)	1.65 (1.57, 1.75)	1.18 (0.92, 1.36)

First 16 days	Original	1.41	2.09	1.15
	
	39%	1.42 (1.38, 1.45)	2.11 (1.97, 2.23)	1.12 (0.83, 1.37)
	
	logistic	1.29 (1.26, 1.32)	2.01 (1.86, 2.17)	1.25 (0.88, 1.56)
	
	58%	1.43 (1.38, 1.48)	2.13 (1.96, 2.30)	1.11 (0.78, 1.43)
	
	logistic	1.11 (1.09, 1.12)	1.60 (1.47, 1.73)	0.90 (0.63, 1.24)
	
	68%	1.43 (1.38, 1.48)	2.14 (1.95, 2.31)	1.10 (0.77, 1.42)
	
	logistic	1.05 (1.05, 1.07)	1.45 (1.34, 1.56)	0.74 (0.52, 1.04)

Key: The first block of results consider the entire outbreak and fit the reproductive number using a four parameter logistic function. The second block consider only the first 16 days where the epidemic is in exponential growth. For each attack rate shown (39%, 58% and 68%) estimates are shown when data missingness is assumed to be constant and when it is assumed to follow a logistic pattern with the initial reporting fraction at 0.4 increasing to 0.9.

**Table 2:** Results with all of the data.

		R_0 _(IQR)	$\hat{\mu }$**(IQR)**	${\hat{\sigma }}^{2}$**(IQR)**
25% pH1N1	Original	1.42	1.96	1.14
	
	39%	1.74 (1.58, 2.14)	2.51 (2.29, 2.73)	1.21 (0.90, 1.49)
	
	logistic	1.44 (1.35, 1.54)	2.38 (2.19, 2.60)	1.32 (1.04, 1.59)
	
	58%	1.76 (1.59, 2.24)	2.47 (2.25, 2.68)	1.31 (0.99, 1.58)
	
	logistic	1.19 (1.14, 1.23)	2.09 (1.91, 2.24)	1.30 (1.02, 1.59)
	
	68%	1.72 (1.55, 2.10)	2.45 (2.24, 2.66)	1.32 (1.01, 1.59)
	
	logistic	1.12 (1.09, 1.15)	1.95 (1.80, 2.12)	1.22 (0.89, 1.54)

50% pH1N1	39%	1.70 (1.53, 2.11)	2.39 (2.18, 2.59)	1.37 (1.08, 1.63)
	
	logistic	1.40 (1.35, 1.46)	2.25 (2.09, 2.41)	1.46 (1.16, 1.70)
	
	58%	1.73 (1.55, 2.17)	2.36 (2.21, 2.55)	1.47 (1.24, 1.68)
	
	logistic	1.15 (1.13, 1.18)	1.93 (1.80, 2.06)	1.33 (1.03, 1.59)
	
	68%	1.62 (1.51, 2.04)	2.34 (2.16, 2.54)	1.38 (1.13, 1.66)
	
	logistic	1.09 (1.07, 1.11)	1.78 (1.66, 1.91)	1.21 (0.91, 1.47)

75% pH1N1	39%	1.61 (1.49 2.05)	2.31 (2.14, 2.51)	1.44 (1.21, 1.68)
	
	logistic	1.38 (1.34, 1.43)	2.18 (2.05, 2.32)	1.52 (1.28, 1.76)
	
	58%	1.92 (1.52, 2.16)	2.32 (2.16, 2.50)	1.51 (1.32, 1.67)
	
	logistic	1.14 (1.12, 1.16)	1.84 (1.73, 1.95)	1.34 (1.07, 1.56)
	
	68%	1.59 (1.48, 2.04)	2.29 (2.12, 2.50)	1.41 (1.18, 1.63)
	
	logistic	1.08 (1.07, 1.10)	1.69 (1.60, 1.80)	1.16 (0.92, 1.42)

Key: The scenarios are as described in the main text of the manuscript and here are broken down into three cases: 25%, 50% or 75% are actually pH1N1.

**Table 3:** Results with the first 16 days of data (i.e. the exponential growth phase).

		R_0 _(IQR)	$\hat{\mu }$**(IQR)**	${\hat{\sigma }}^{2}$**(IQR)**
25% H1N1	Original	1.41	2.09	1.15
	
	39%	1.45 (1.33, 1.59)	2.36 (2.06, 2.66)	0.97 (0.59, 1.42)
	
	logistic	1.34 (1.28, 1.42)	2.28 (2.00, 2.54)	1.17 (0.80, 1.19)
	
	58%	1.45 (1.33, 1.56)	2.26 (1.93, 2.57)	1.04 (0.65, 1.48)
	
	logistic	1.14 (1.11, 1.18)	1.87 (1.68, 2.09)	1.02 (0.73 (1.45)
	
	68%	1.45 (1.36, 1.55)	2.28 (2.04, 2.54)	1.06 (0.66, 1.48)
	
	logistic	1.08 (1.06, 1.11)	1.72 (1.53, 1.92)	0.92 (0.61, 0.70)

50% H1N1	39%	1.46 (1.38, 1.53)	2.27 (2.02, 2.47)	1.03 (0.68, 1.52)
	
	logistic	1.31 (1.27, 1.37)	2.15 (1.91, 2.34)	1.21 (0.80, 1.61)
	
	58%	1.41 (1.33, 1.50)	2.10 (1.80, 2.41)	1.18 (0.74, 1.57)
	
	logistic	1.12 (1.10, 1.15)	1.74 (1.57, 1.90)	0.96 (0.68, 1.40)
	
	68%	1.45 (1.39, 1.52)	2.23 (2.02, 2.42)	1.08 (0.74, 1.51)
	
	logistic	1.07 (1.05, 1.08)	1.57 (1.42, 1.73)	0.85 (0.57, 1.19)

75% H1N1	39%	1.43 (1.38, 1.49)	2.16 (1.97, 2.37)	1.10 (0.75, 1.48)
	
	logistic	1.30 (1.26, 1.34)	2.07 (1.87, 2.25)	1.19 (0.83, 1.56)
	
	58%	1.37 (1.32, 1.42)	1.93 (1.73, 2.18)	1.16 (0.74, 1.53)
	
	logistic	1.11 (1.09, 1.13)	1.65 (1.49, 1.79)	0.93 (0.64, 1.29)
	
	68%	1.44 (1.38, 1.50)	2.17 (1.97, 2.36)	1.03 (0.73, 1.43)
	
	logistic	1.06 (1.05, 1.07)	1.49 (1.37, 1.62)	0.77 (0.51, 1.08)

Key: The scenarios are as described in the main text of the manuscript and here are broken down into three cases: 25%, 50% or 75% are actually pH1N1.

When all of the data are considered, the results are similar. Without adjustment for missingness R_0 _is estimated to be 1.42 and the mean of the serial interval is 1.96 days with a variance of 1.14. Under the constant missingness scheme the reproductive number estimates increase with the attack rate (between 1.49 and 1.54), as well as the mean of the serial interval (2.15-2.25 days). When the reporting fraction increases through time, the reproductive number is smaller and decreases as the AR increases (1.29 to 1.05). The mean of the serial interval follows a similar pattern ranging between 2.01 for AR = 39% and 1.45 for AR = 68%. The results from the overreporting scenarios follow a similar pattern, assuming that the amount of overreporting is consistent throughout the epidemic. The results indicate that if reporting were in fact increasing throughout the epidemic, then the original results could be overstating the magnitude of both the reproductive number and the mean of the serial interval.

Table 4 provides the results using the method of Wallinga and Teunis [3] and the exponential growth model to estimate R_0_. The estimates using the MLE estimator of the serial interval (i.e. with mean of 1.96 for WT or 2.09 for exponential) are comparable to those obtained with the MLE method and are 1.48 for the WT method and 1.40 for the exponential method compared to 1.41 for the MLE method. Those with the Fraser et al [16] estimate (mean SI = 1.91 days) tend to differ as a direct function of the mean SI (WT R_0 _= 1.57, exponential R_0 _= 1.36). Overall the impact of missing data is the same, regardless of the estimation method used.

**Table 4:** Results with Wallinga Teunis estimator 3 (R0 is obtained by averaging over the first 16 days).

	Wallinga & Teunis R_0 _Estimate (IQR)		Exponential R_0 _estimate (IQR)		
	**MLE SI estimate**	**Fraser et al SI**	**MLE SI estimate**	**Fraser et al SI**	**Growth rate (r)**

Original	1.48	1.57	1.40	1.36	0.19

39%	1.72 (1.60, 1.91)	1.86 (1.70, 2.18)	1.30 (1.26, 1.35)	1.28 (1.23, 1.32)	0.15 (0.12, 0.15)

logistic	1.35 (1.25, 1.37)	1.39 (1.27, 1.53)	1.06 (1.02, 1.11)	1.06 (1.02, 1.10)	0.03 (0.01, 0.05)

58%	1.55 (1.49, 1.63)	1.64 (1.57, 1.73)	1.39 (1.37, 1.41)	1.36 (1.34, 1.38)	0.19 (0.18, 0.20)

logistic	1.19 (1.16, 1.22)	1.22 (1.18, 1.26)	1.08 (1.06, 1.11)	1.08 (1.06, 1.08)	0.04 (0.03, 0.05)

68%	1.59 (1.51, 1.70)	1.68 (1.59, 1.80)	1.38 (1.36, 1.41)	1.35 (1.33, 1.35)	0.18 (0.17, 0.19)

*logistic*	1.20 (1.16, 1.22)	1.24 (1.19, 1.29)	1.08 (1.06, 1.11)	1.08 (1.05, 1.10)	0.04 (0.03, 0.05)

## Discussion

We have shown the impact of reporting issues on estimates of the reproductive number and the serial interval. Using a MLE based method, we show that the estimate of the reproductive number is unaffected if the reporting fraction is constant through time. However, if the amount of reporting increases, then we will overestimate the reproductive number if no adjustment is made. The converse is true should reporting tend to decrease over time. The simulation results and the work from La Gloria tend to support this theoretical result. We note that using other methods of estimation of the reproductive number (Wallinga and Tuenis [3] and exponential growth) yield the same trends.

From our simulation work, we notice that the mean of the serial interval appears to follow the trend of the reproductive number. For instance, if the reproductive number is overestimated, then the mean of the serial interval tends to be too large, as well. Thus missing data not only impacts the estimate of the reproductive number, but also the estimation of the serial interval.

We additionally note several caveats to the work presented here. First, we have assumed homogenous mixing by not accounting for any variability in the disease parameters among subgroups. Clearly disease outbreaks are dynamic and impacted by multiple factors in the effected population. We follow the precedent commonly used and assume homogeneity in the population. It is not clear how much results might change if this assumption is relaxed.

Second, we take a frequentist approach and do not allow for variability in the parameters that are assumed known [15] recently published work allowing for a Bayesian approach to estimation where prior information on the serial interval can be incorporated into estimation. The values for the end results are likely to not vary significantly, if the prior information is not informative, there is sufficient data for estimation, or the serial interval is longer than one day. However there are cases where this prior information can improve the estimates and might impact the conclusions drawn here. In our setting we assume that there is no prior information, or if there is, such as the serial interval being known, that it is known with certainty. In this case, this assumption might lead to overestimating of the mean of the serial interval.

## Conclusions

Failure to account for ascertainment of cases can have a substantial impact on the estimation of key epidemiological parameters. If the fraction of cases reported does not change dramatically throughout the course of the epidemic, then estimates may not be impacted substantially. When the reporting fraction varies through the course of an epidemic, as it likely will, estimates can be substantially impacted. It is important that epidemiological studies of infectious disease outbreak seek to account and better understand the nature of reporting and make appropriate adjustments in the methods used to obtain results. We have shown how this can be done for the MLE method and illustrated its use for recent Influenza A/H1N1 outbreak data from La Gloria, Mexico. In that case results were off by as much as 34% when underreporting was not considered.

Data do exist, however, to obtain an idea of the level of underreporting [13] use information on hospital admissions in the current H1N1 pandemic to obtain an estimate of the degree of underreporting. Further [15] has recently shown that incorporating contact tracing data into the estimation of the serial interval in a Bayesian framework can improve estimates. A similar approach could be used to improve estimates to account for suspected levels of misreporting. Clearly the need exists to use innovative methods to ascertain reporting issues in data using existing data, or by the collection of additional data or well-planned studies that can be rapidly initiated in the event of an outbreak. For instance, one might consider carefully studying a smaller population to determine the rate of reporting there. This could inform the overall rate of reporting. At the least, estimates should be reported as ranges, rather than as a single point estimate to indicate plausible values for the estimate.

## APPENDIX

### Proof of Proposition 1

It is straightforward to observe from the definition of ${\tilde{R}}_{0}$ that when *q_t _*= *q*, the correct estimator simplifies to

$${\tilde{R}}_{0}=\frac{\sum_{t=1}^{T}M_{t}}{\sum_{t=1}^{T}\sum_{j=1}^{k}p_{j}M_{t-j}}.$$

In this case ${\hat{R}}_{0}={\tilde{R}}_{0}$, indicating that estimation of R_0 _is not impacted by the missingness in the data.

### Proof of Proposition 2

Without loss of generality, we consider the simple branching process estimator, where *p*_1 _= 1 and *p_j _*= 0 if j > 1. Additionally let *q*_2 _= *rq*_1_, where r > 1. Then the estimator with missingness taken into consideration becomes

$$\begin{array}{l} {\tilde{R}}_{0}=\frac{\frac{1}{q_{1}}\sum_{t=1}^{t_{c}}M_{t}+\frac{1}{rq_{1}}\sum_{t=t_{c}+1}^{T}M_{t}}{\frac{1}{q_{1}}\sum_{t=1}^{t_{c}+1}M_{t-1}+\frac{1}{rq_{1}}\sum_{t=t_{c}+2}^{T}M_{t-1}}\\ =\frac{r\sum_{t=1}^{t_{c}}M_{t}+\sum_{t=t_{c}+1}^{T}M_{t}}{r\sum_{t=1}^{t_{c}+1}M_{t-1}+\sum_{t=t_{c}+2}^{T}M_{t-1}} \end{array}$$

Now consider the difference ${\tilde{R}}_{0}-{\hat{R}}_{0}$.

$$\begin{array}{l} {\tilde{R}}_{0}-{\hat{R}}_{0}=\frac{r\sum_{t=1}^{t_{c}}M_{t}+\sum_{t=t_{c}+1}^{T}M_{t}}{r\sum_{t=1}^{t_{c}+1}M_{t-1}+\sum_{t=t_{c}+2}^{T}M_{t-1}}-\frac{\sum_{t=1}^{T}M_{t}}{\sum_{t=1}^{T}M_{t-1}}.\\ =\left(\frac{1}{\sum_{t=1}^{T}M_{t-1}(r\sum_{t=1}^{t_{c}+1}M_{t-1}+\sum_{t=t_{c}+2}^{T-1}M_{t-1})}\right)\times \\ (rM_{0}\sum_{t=1}^{t_{c}}M_{t}+r\sum_{t=1}^{t_{c}}M_{t}\sum_{t=1}^{T-1}M_{t}+\\ M_{0}\sum_{t=t_{c}+1}^{T}M_{t}\sum_{t=1}^{T-1}M_{t}-r\sum_{t=1}^{T}M_{t}\sum_{t=1}^{t_{c}}M_{t}-\\ rM_{0}\sum_{t=1}^{T}M_{t}-\sum_{t=t_{c}+1}^{T-1}M_{t}\sum_{t=1}^{T}M_{t}).\\ =\frac{\left(1-r)(M_{T}\sum_{t=1}^{t_{c}}M_{t}+M_{0}\sum_{t=t_{c}+1}^{T}M_{t}\right)}{\sum_{t=1}^{T}M_{t-1}(r\sum_{t=1}^{t_{c}+1}M_{t-1}+\sum_{t=t_{c}+2}^{T-1}M_{t-1})}<0. \end{array}$$

### Results for the standard error of the reproductive number

We now illustrate the impact of reporting issues on the standard error of the estimators, ${\hat{R}}_{0}$ and ${\tilde{R}}_{0}$ using the formula provided in [21] and given by

$$SE({\hat{R}}_{0})={\left(\frac{{\hat{R}}_{0}}{\sum_{t=1}^{T}N_{t-1}}\right)}^{1/2}.$$

**Proposition A1**. If *q_t _*= *q *< 1 for all values of t, then SE(${\hat{R}}_{0}$) < SE(${\tilde{R}}_{0}$), i.e. we underestimate the SE. If *q_t _*= *q *> 1 then SE(${\hat{R}}_{0}$) > SE(${\tilde{R}}_{0}$), i.e. we tend to overestimate the standard error.

**Proposition A2**. If *q_t _*= *q*_1_, *t *≤ *t_c _*and *q_t _*= *q*_2_, *t *>*t_c _*then

i. If *q*_1 _<*q*_2 _< 1 then $SE({\hat{R}}_{0})>SE({\tilde{R}}_{0})$.

ii. If *q*_1 _>*q*_2 _> 1 then $SE({\hat{R}}_{0})<SE({\tilde{R}}_{0})$.

iii. If *q*_2 _>*q*_1 _> 1 or *q*_2 _<*q*_1 _< 1 then the results for the SE are not clearly defined.

### Results for overreporting in La Gloria

Following are the results when we consider that only a fraction of the total cases in La Gloria were actually of the pH1N1 strain. For this scenario, we simulate 1000 datasets, as before. These datasets are interpreted as providing the total number of ILI cases. Of these, we assume that only a proportion is actually of the pandemic strain. We allow this to be 0.25, 0.50, or 0.75. Cases are randomly selected as pH1N1 cases and included in the analysis. Results are given in Tables 2 and 3.

## Competing interests

The authors declare that they have no competing interests.

## Authors' contributions

LFW and MP conceived the project and were involved in drafting the manuscript. LFW carried out the simulations and analysis. Both authors read and approved the final manuscript.
